# Inhibition of Karyopherin-α2 Augments Radiation-Induced Cell Death by Perturbing BRCA1-Mediated DNA Repair

**DOI:** 10.3390/ijms20112843

**Published:** 2019-06-11

**Authors:** Kyung-Hee Song, Seung-Youn Jung, Jeong-In Park, Jiyeon Ahn, Jong Kuk Park, Hong-Duck Um, In-Chul Park, Sang-Gu Hwang, Hunjoo Ha, Jie-Young Song

**Affiliations:** 1 Division of Radiation Biomedical Research, Korea Institute of Radiological & Medical Sciences, Seoul 01812, Korea; songkh@kirams.re.kr (K.-H.S.); unicerty@gmail.com (S.-Y.J.); jipark@kirams.re.kr (J.-I.P.); ahnjy@kirams.re.kr (J.A.); jkpark@kirams.re.kr (J.K.P.); hdum@kirams.re.kr (H.-D.U.); parkic@kirams.re.kr (I.-C.P.); sgh63@kirams.re.kr (S.-G.H.); 2 Graduate School of Pharmaceutical Sciences, College of Pharmacy, Ewha Womans University, Seoul 03760, Korea

**Keywords:** ionizing radiation, karyopherin-α2, DNA repair, radioresistance, BRCA1

## Abstract

Ionizing radiation (IR) has been widely used in the treatment of cancer. Radiation-induced DNA damage triggers the DNA damage response (DDR), which can confer radioresistance and early local recurrence by activating DNA repair pathways. Since karyopherin-α2 (KPNA2), playing an important role in nucleocytoplasmic transport, was significantly increased by IR in our previous study, we aimed to determine the function of KPNA2 with regard to DDR. Exposure to radiation upregulated KPNA2 expression in human colorectal cancer HT29 and HCT116 cells and breast carcinoma MDA-MB-231 cells together with the increased expression of DNA repair protein BRCA1. The knockdown of *KPNA2* effectively increased apoptotic cell death via inhibition of BRCA1 nuclear import following IR. Therefore, we propose that KPNA2 is a potential target for overcoming radioresistance via interruption to DDR.

## 1. Introduction

Radiotherapy (RT), the most common therapeutic modality for cancers, is used to treat up to 50% of patients with cancer [1]. However, a considerable number of patients undergoing RT acquires radioresistance and shows recurrence, with consequent worsening of the prognosis and shortening of survival. The exploration of the molecular mechanisms of radiation resistance and the development of effective prognostic markers are extremely important to decide whether or not to treat patients with RT [2].

Many mechanisms have been proposed to be responsible for radiation resistance, including adaptive response, DNA damage repair, adhesion, inflammation, hypoxia, and other survival signaling pathways [3]. Ionizing radiation (IR) induces several types of cancer cell death such as apoptosis, necrosis, mitotic catastrophe, autophagy, and senescence through DNA damage as well as radiolytic products such as free radicals. However, IR by itself does not effectively destroy high-grade, aggressive cancers because of the ability of tumor cells to initiate a DNA damage response (DDR) by activating multiple DNA damage sensing and repair pathways [4]. For example, although RT is the most common treatment against glioblastoma, as glioma stem cells possess a highly efficient DNA repair system resulting in the development of radioresistance, glioblastoma is the most fatal and incurable human cancer [5]. Many DNA repair factors have been isolated and extensively studied: they include DNA damage sensors (ATM, ATR, checkpoint kinase [Chk]1, Chk2, p53), base or nucleotide excision repair molecules (apurinic/apyrimidinic [AP] endonuclease [APE-1], X-ray repair cross-complementing 1 [XRCC1], excision repair cross-complementation group 1 [ERCC1], xeroderma pigmentosum group D [XPD]), non-homologous end-joining (Ku70/Ku80, DNA-dependent protein kinase catalytic subunit [DNA-PKcs]) factors, and homologous recombination (RAD51, breast cancer gene 1 [BRCA1], H2AX, Bloom syndrome protein [BLM], phosphatase and tensin homolog [PTEN]) factors [6]. Targeting DDR signaling factors and repair pathways in cancer may provide a therapeutic advantage to overcome radiation resistance.

In a previous study, we identified karyopherin-α2 (KPNA2, also called importin-α1) as a protein upregulated in human colorectal cancer HT29 cells exposed to single or fractionated IR [7]. There are seven previously described human importin-α forms, each encoded by a different gene. Although some functional redundancy exists, importin-α family members show preferences for specific types of nuclear localization sequence (NLS) cargo, which is important for controlling differentiation pathways [8]. KPNA2 is a member of the karyopherin superfamily that is involved in transporting molecules between the cytoplasm and the nucleus. Specifically, KPNA2 binds to the NLS of its cargo proteins and to karyopherin-β1, and the whole protein complex translocates across the nuclear envelope through the nuclear pore complex [9]. KPNA2 interacts with a variety of macromolecules or other proteins that are associated with cellular maintenance, transcriptional regulation, carcinogenesis, aberrant activation of differentiation pathways, viral induction, and immune response [10]. Dysregulation of the nuclear transport system causes localization shifts of specific cargo proteins, leading to pathophysiological alterations by their incorrect spatiotemporal arrangement. Therefore, the inhibition of nuclear transport machinery—as well as targeted mislocalized proteins—has been a promising strategy to develop therapeutic intervention. To date, several nuclear import/export inhibitors have been developed and tested their anticancer or antiviral potentials in clinical trials [11].

Many studies have reported that KPNA2 is highly expressed in diverse types of cancer, including breast cancer, gastric cancer, hepatocarcinoma, lung cancer, melanoma, and ovarian cancer [12,13,14,15,16,17,18]. In addition, a strong correlation between its overexpression and poor prognosis in patients with various cancers has been reported. KPNA2 exerts this effect by influencing the nuclear import of molecules that are involved in cell proliferation, differentiation, DNA repair, and tumorigenesis [13,19,20]. Therefore, it has been hypothesized that KPNA2 is a novel target related to resistance against cancer therapy.

In this study, we aimed to investigate how radiation increases the levels of KPNA2 in tumor cells and how KPNA2 plays a role in radioresistance.

## 2. Results

### 2.1. KPNA2 is Associated with Radioresistance in Human Colorectal Cancer Cells

**Figure 1:**
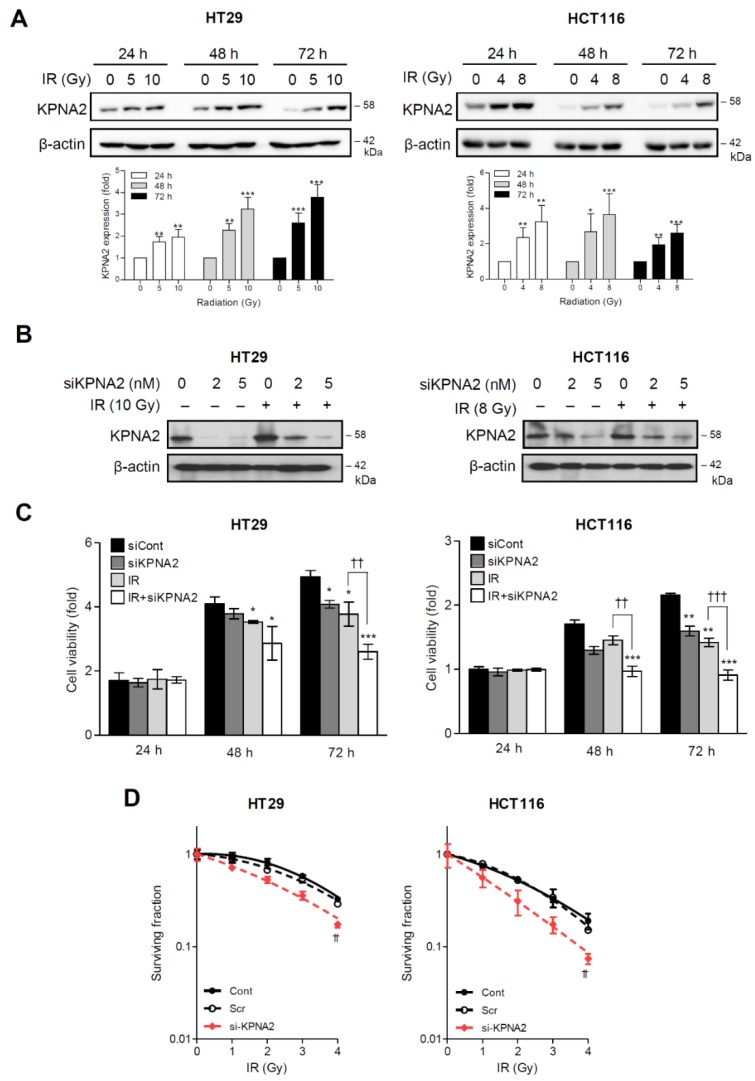
Inhibition of radiation-induced KPNA2 expression increases the radiosensitivity of human colorectal cancer cells. (**A**) The levels of KPNA2 in HT29 and HCT116 cells were determined by western blot analysis at the indicated times following ionizing radiation (IR). (**B**) Cells were transfected with *KPNA2* siRNA (siKPNA2) for 24 h. The reduced expression of KPNA2 was confirmed by western blotting. (**C**) The cell viability of control siRNA (siCont, 5 nM)- or siKPNA2 (5 nM)-transfected cells with or without treatment with IR was measured by 3-(4,5-dimethylthiazol-2-yl) -2,5-diphenyltetrazolium bromide (MTT) assay at the indicated times. * *p* < 0.05, ** *p* < 0.01, *** *p* < 0.001 vs. control siRNA-transfected group, ^††^ *p* < 0.01, ^†††^ *p* < 0.001 vs. irradiated group. (**D**) The clonogenic survival fraction was evaluated in HT29 or HCT116 cells transfected with control or *KPNA2* siRNA for 24 h following IR. The data show representative results and are presented as mean ± SD of three independent experiments. ^††^ *p* < 0.01 vs. control siRNA-transfected group.

We detected a marked induction of KPNA2 expression upon IR exposure in colorectal cancer HT29 and HCT116 cells in a time- and dose-dependent manner (Figure 1A). Because of the tight association between high levels of KPNA2 expression in cancer cells and resistance to anticancer therapy [21], we hypothesized that radiation-induced KPNA2 expression attenuates the efficacy of IR. To investigate this hypothesis, we silenced the expression of *KPNA2* in HT29 and HCT116 cells by transfecting small interfering RNA (siRNA; Figure 1B) and evaluated cell viability. Knockdown of *KPNA2*, obtained with siRNA targeting *KPNA2* (siKPNA2), inhibited the growth of irradiated cells, compared with the non-irradiated ones; this effect was similar in both cell lines (Figure 1C). Additionally, the clonogenic survival of *KPNA2*-depleted cells was significantly decreased in response to radiation (Figure 1D), suggesting that KPNA2 plays an important role in the acquisition of radioresistance.

### 2.2. Knockdown of KPNA2 Increases Radiation-Induced Apoptosis

**Figure 2:**
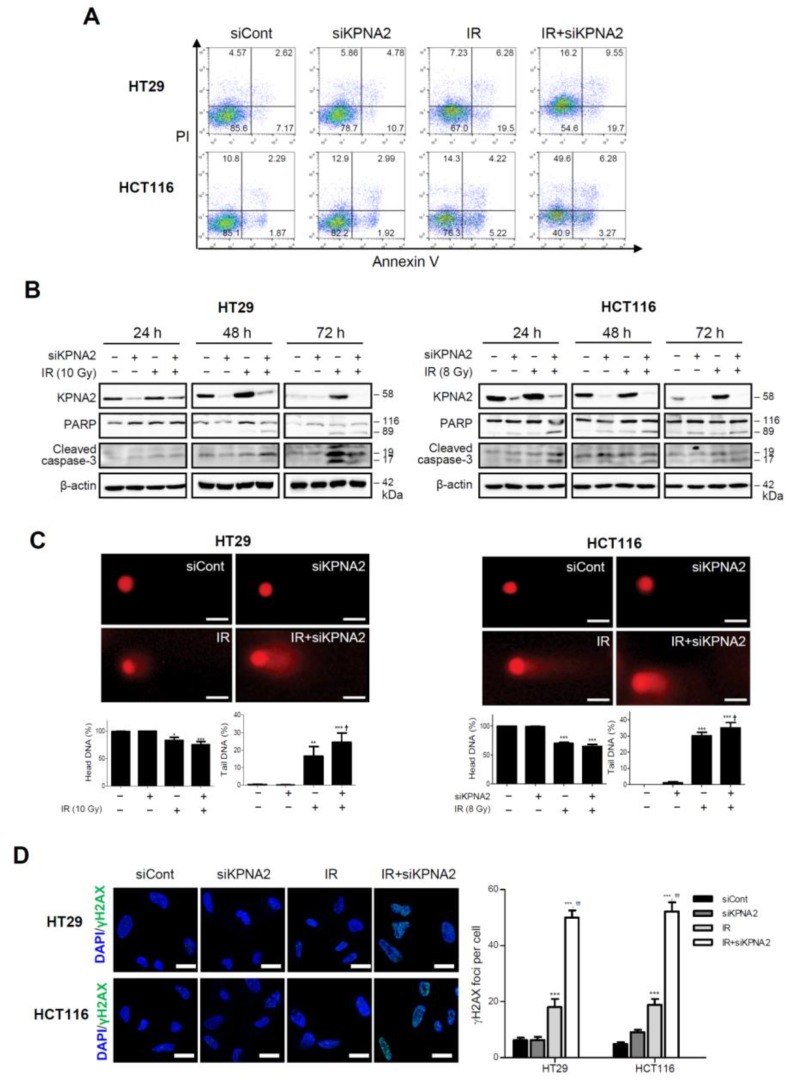
*KPNA2* silencing increases apoptosis and DNA damage. (**A**) The effect of the knockdown of *KPNA2* on apoptotic cell death was measured by flow cytometry. Cells were transfected with control siRNA (siCont) or *KPNA2*-specific siRNA (siKPNA2) for 24 h and then exposed to 8 or 10 Gy of IR. After 72 h, cell death was determined by annexin V allophycocyanin (APC) and propidium iodide (PI) staining and flow cytometry. (**B**) *KPNA2*-depleted cells with or without IR were analyzed for the levels of PARP and cleaved caspase-3 by western blotting at the indicated times. Results are representative of three independent experiments. (**C**) Fluorescence microscopy visualization of comet assays shows the levels of DNA damage in *KPNA2*-depleted HT29 and HCT116 cells 48 h after IR (original magnification ×100, scale bars 20 μm). The percentage of head and tail DNA content was quantified in 100 comet images. Results represent the mean ± SD of three independent experiments. * *p* < 0.05, ** *p* < 0.01, *** *p* < 0.001 vs. control siRNA-transfected group, ^†^ *p* < 0.05 vs. irradiated group. (**D**) Immunofluorescence staining shows the expression of γH2AX (green) in *KPNA2*-depleted HT29 and HCT116 cells 48 h after IR (original magnification ×630, scale bars 20 μm). The corresponding bar charts show the quantification of γH2AX foci per cell. Data are the mean ± SD of three independent experiments. *** *p* < 0.001 vs. control siRNA-transfected group, ^†††^ *p* < 0.001 vs. irradiated group.

To investigate the molecular mechanism of radioresistance mediated by KPNA2, we determined whether apoptotic cell death is increased by *KPNA2* depletion in irradiated cells using the annexin V/propidium iodide (PI) staining method. As shown in Figure 2A, a significant induction of apoptosis was observed in siKPNA2-treated and irradiated cells compared with control or irradiated cells (45.4% vs. 14.4% or 33.0% in HT29 cells, and 59.1% vs. 14.9% or 23.7% in HCT116 cells, respectively). Western blotting revealed that the levels of apoptotic markers, including cleaved poly-(ADP-ribose) polymerase (PARP) and caspase-3, significantly increased in *KPNA2*-silenced HT29 cells 48 h and, more so, 72 h after radiation (Figure 2B). Similar changes were observed earlier in HCT116 cells, suggesting that HCT116 cells are more sensitive to IR than HT29 cells and that KPNA2-mediated function may not be restricted by cell type or molecular characteristics such as p53 status.

We next studied whether *KPNA2* silencing enhanced DNA damage by radiation using comet assay, a sensitive and reliable tool to evaluate the presence and levels of DNA strand breaks [22]. siKPNA2- and IR-treated cells showed increased DNA damage 48 h after radiation compared with control or IR-treated cells, as indicated by higher fluorescence intensity in their comet tails (Figure 2C; 24.44% tail DNA in HT29 cells and 35.04% in HCT116 cells, respectively). Cells were subsequently analyzed for the induction of DNA damage using γH2AX as a marker for double-strand breaks (DSBs). Similar to the results of comet assay, combined *KPNA2* depletion with IR generated more γH2AX foci than treatment with IR alone (Figure 2D). These data demonstrated that *KPNA2* silencing enhances radiation-induced apoptotic cell death by increasing DNA damage.

### 2.3. Knockdown of KPNA2 Prevents BRCA1 Activation

**Figure 3:**
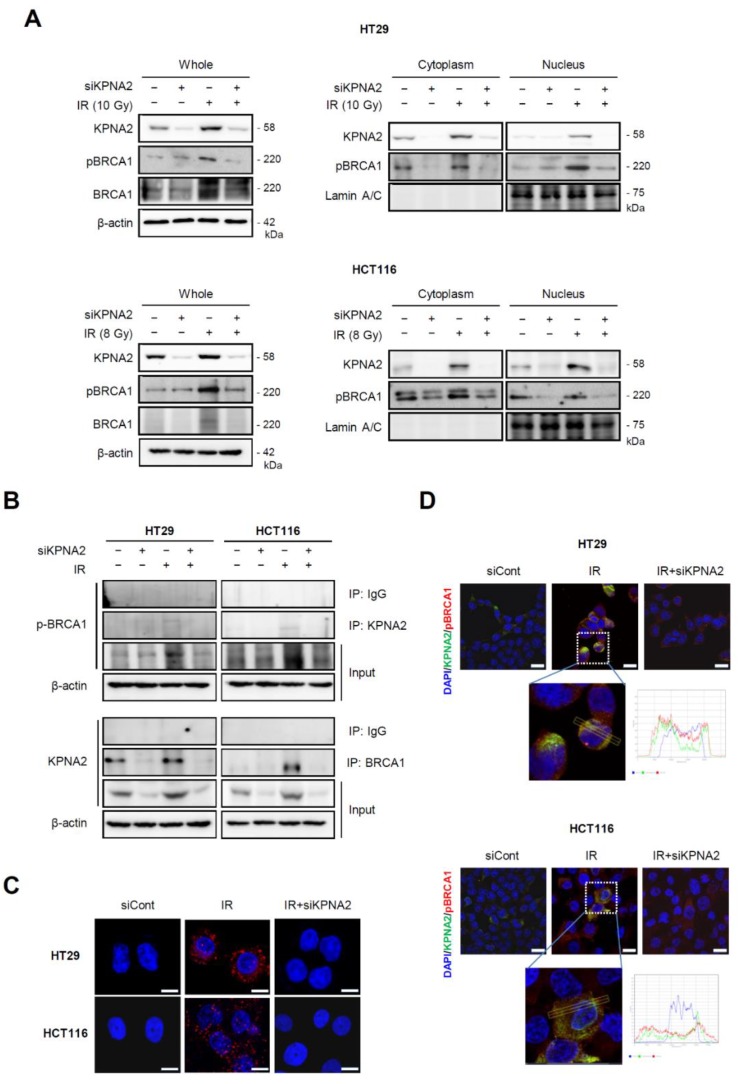
*KPNA2* depletion interferes with the activation of radiation-induced BRCA1 proteins. HT29 and HCT116 cells were transfected with *KPNA2* siRNA for 24 h and then treated with IR for 48 h. (**A**) Whole lysates and nuclear and cytoplasmic fractions of cells were analyzed by western blot for the indicated proteins. (**B**) Immunoblot analysis of KPNA2-BRCA1 interaction in HT29 and HCT116 cells pre-treated as indicated and immunoprecipitated for anti-KPNA2 or BRCA1. (**C**) Cells were fixed and incubated with mouse anti-KPNA2 antibody together with rabbit anti-BRCA1 antibody, followed by in situ proximity ligation assay (PLA). DNA was stained with 4′,6-diamidino-2-phenylindole (DAPI). Representative confocal images are shown; each red dot represents a single interaction between KPNA2 and BRCA1 (original magnification ×400, scale bars 20 μm). (**D**) Immunofluorescence staining showing the localization of KPNA2 (green) and pBRCA1 (red) in *KPNA2*-depleted HT29 and HCT116 cells 48 h after IR (original magnification ×630, scale bars 20 μm). The peak graph represents the localization of KPNA2 (green line) and pBRCA1 (red line) in the nucleus (blue line).

KPNA2 could affect cell proliferation and DDR through the translocation of several proteins from the cytoplasm to the nucleus. As it is well known that BRCA1 becomes rapidly activated in response to DNA damage by hyperphosphorylation at multiple sites by several kinases including ATM and Chk2 [23,24], we determined the levels of BRCA1 phosphorylation. Along with the upregulation of KPNA2 by IR, the expression levels of pBRCA1 also increased in both cell lines. In addition, this protein was markedly reduced in the nuclei of *KPNA2*-depleted and irradiated cells (Figure 3A). These results indicated that radiation-induced KPNA2 contributes to the nuclear localization of BRCA1.

To determine the interaction between KPNA2 and BRCA1, immunoprecipitation (IP) experiments were performed. When both cells were immunoprecipitated with anti-α-BRCA1, the KPNA2-BRCA1 interaction was clearly shown by immunoblots. In addition, IP with anti-α-KPNA2 revealed that endogenous levels of KPNA2 rarely interact with pBRCA1, but this interaction was increased by irradiation (Figure 3B). We further confirmed the interaction between KPNA2 and BRCA1 by proximity ligation assay (PLA), which enables the visualization and quantification of specific protein-protein interaction events in situ. As shown in Figure 3C, the binding of KPNA2 and BRCA1 was induced by IR (red fluorescence); the KPNA2/BRCA1 complex was located in both the cytoplasm and nucleus of irradiated cells, whereas it was not identified in *KPNA2*-depleted cells treated with IR. Furthermore, KPNA2/BRCA1 co-localization was enhanced by IR in the cytoplasm as well as in the nuclei but hardly detected in *KPNA2*-depleted and irradiated cells based on double fluorescent immunostaining (Figure 3D). Phosphorylated BRCA1 was predominantly located in the perinuclear region and cytoplasm of *KPNA2*-depleted and irradiated cells. These data indicated that radiation-induced KPNA2 favors the transport of BRCA1 into the nucleus and activates DNA repair, leading to the development of radiation resistance.

### 2.4. AMP-Activated Protein Kinase (AMPK) Regulates KPNA2 Expression

**Figure 4:**
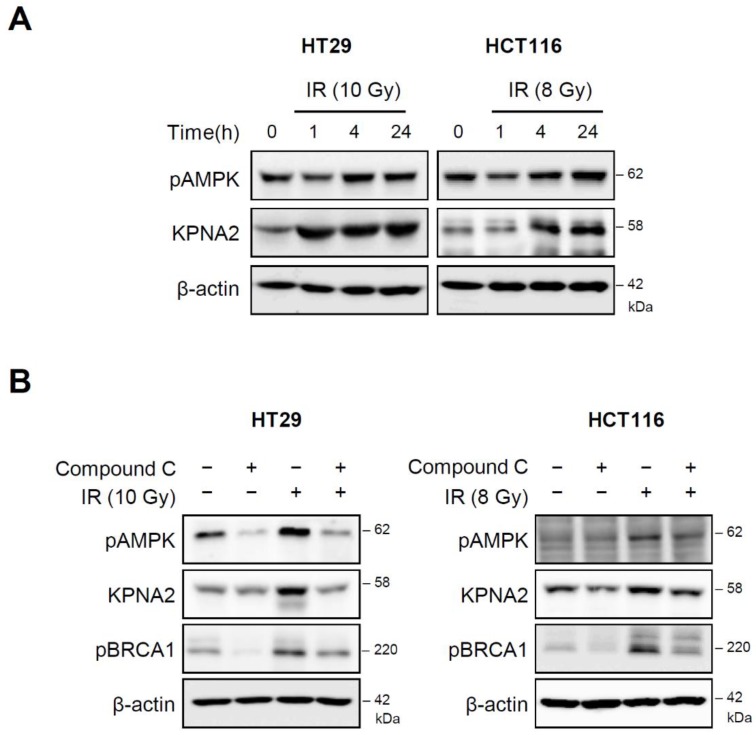
Radiation-induced AMPK regulates KPNA2. (**A**) The expression of pAMPK and KPNA2 in HT29 and HCT116 cells was measured by western blot analysis at the indicated times after IR. (**B**) HT29 and HCT116 cells were treated with an AMPK inhibitor, Compound C (10 μM), for 1 h prior to IR. After further incubation for 8 h, the levels of indicated proteins were analyzed by western blotting. Results are representative of three independent experiments.

Next, we investigated the molecular mechanisms that induced KPNA2 expression. Previous reports have shown that AMPK activation by polyamine or radiation increases KPNA2 levels for the regulation of the cell cycle and survival [25]. Therefore, we investigated whether, in our system, radiation induced KPNA2 through the activation of AMPK. We found that radiation increased the phosphorylation of AMPK together with KPNA2 levels (Figure 4A). To confirm that the radiation-induced AMPK activation regulates KPNA2 levels, we treated both cell lines with Compound C (an AMPK inhibitor). The radiation-mediated AMPK phosphorylation was blocked, and the upregulation of KPNA2 and BRCA1 levels by radiation was decreased upon Compound C treatment (Figure 4B). These results suggested that AMPK activation might play a role in the expression and activity of KPNA2 upon radiation.

### 2.5. KPNA2-Depleted Breast Cancer Cells Exhibit Radiosensitivity

Microarray and immunohistochemistry assays have shown that KPNA2 expression is higher in several cancer specimens, such as breast, colon, lung, oral, pancreatic, and gastric cancers, compared with adjacent normal tissue [13]. Therefore, we further investigated whether *KPNA2* silencing induces apoptosis of breast cancer MDA-MB-231 cells upon radiation and disturbs the nuclear transport of BRCA1 molecules.

**Figure 5:**
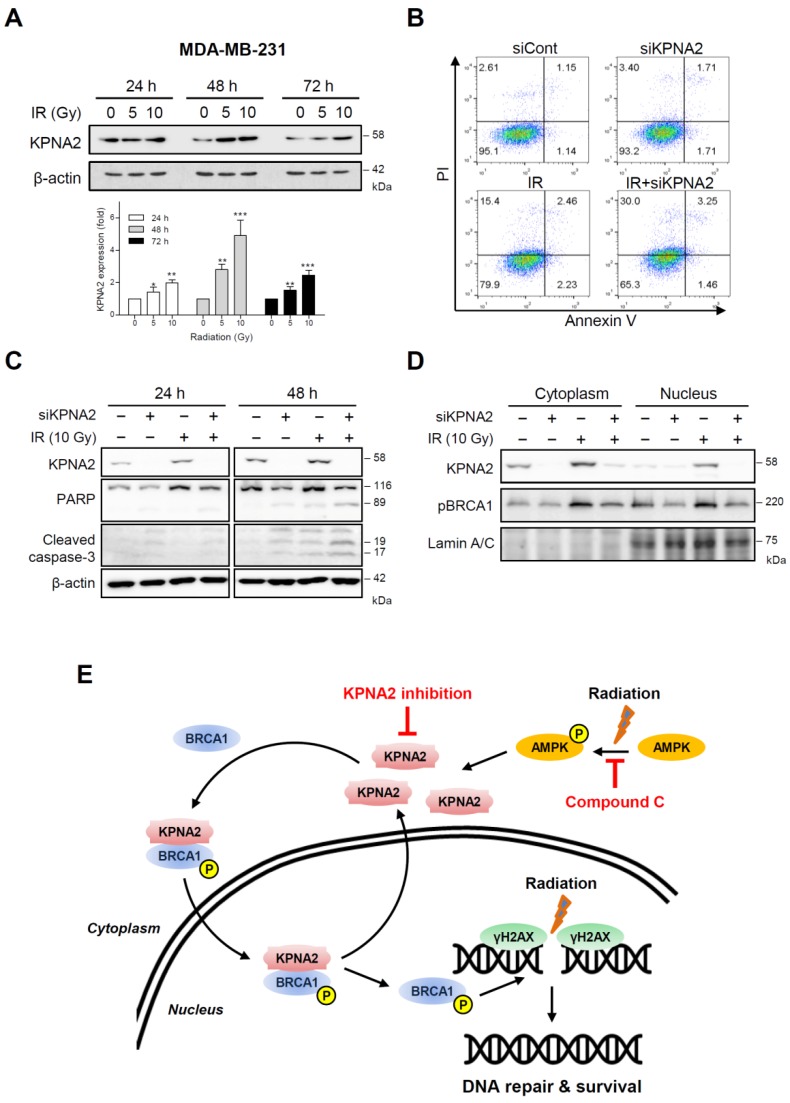
Inhibition of KPNA2 increases radiation-induced cell death in MDA-MB-231 cells. (**A**) MDA-MB-231 cells were lysed at the indicated times after exposure to 5 or 10 Gy of IR and subjected to western blot analysis for the detection of KPNA2 levels. (**B**) Cells were transfected with *KPNA2*-specific siRNA (5 nM) for 24 h prior to IR with 10 Gy. Apoptotic cell death of *KPNA2*-depleted cells with or without IR was measured by annexin V/PI staining using flow cytometry. (**C**,**D**) The expression of apoptosis-related factors (PARP, cleaved caspase-3) and pBRCA1 was detected by western blot analysis. Results are representative of three independent experiments. (**E**) Schematic model of the potential interaction of KPNA2 and BRCA1. DNA damage caused by IR increases the level of KPNA2 through AMPK activation concomitantly with the induction of BRCA1. Increased KPNA2 directly binds to BRCA1 and transports it from the cytoplasm to the nucleus. Therefore, KPNA2 appears to play a major role in the nuclear import of critical cargo proteins that regulate cell proliferation and survival and can be used as a marker for poor prognosis with radiation therapy.

Consistently, KPNA2 expression was increased in MDA-MB-231 cells exposed to radiation in a time- and dose-dependent manner (Figure 5A). *KPNA2* silencing increased radiation-mediated cell death by about 34.7%, while radiation alone increased apoptosis by about 20.1% (Figure 5B). Additionally, the protein levels of PARP and cleaved caspase-3 were increased in *KPNA2*-depleted and irradiated cells, in agreement with the above results (Figure 5C). The depletion of *KPNA2* reduced the expression of pBRCA1, which was increased by radiation (Figure 5D). These data indicated that the cancer-specific marker KPNA2 could be a valuable target to overcome resistance against RT via regulation of DDR signaling.

## 3. Discussion

IR exerts its cytotoxic effect by the induction of DSBs and non-DSB in highly clustered DNA lesions consisting of a combination of single-strand breaks (SSBs), abasic sites, and oxidized bases within 5–10 base pairs [26] that lead to chromosomal aberrations [27,28], cell death [29], and accumulation in tumor tissues [30]. RT, a major therapeutic modality for cancer, kills cancer cells by damaging DNA [31]. Leukemia and germ cell tumors, composed of rapidly growing cells, are sensitive to RT and effectively killed by modest doses of radiation. However, some types of cancers are notably radioresistant and require considerably higher doses of radiation or combination therapy with chemotherapy and/or immunotherapy to achieve sufficient therapeutic outcomes [32]. The differential radiosensitivity may depend on many factors, such as the DNA damage repair process, cell cycle regulation, survival, and growth signal transduction pathways; angiogenic factors; hypoxic conditions; and cellular metabolic pathways [33].

In our previous study, using quantitative proteomic analysis, we identified KPNA2 as one of the proteins whose levels are significantly increased by single or fractionated radiation in HT29 cells and that cause the activation of immune cells [7]. KPNA2 has been proposed to regulate cell proliferation, differentiation, migration, and DNA repair [16,17,20]. In addition, it has been documented that KPNA2 expression in tumors is a powerful predictive factor of poor prognosis in patients [10,18,21]. Recently, it has been suggested that cellular stresses, including oxidative stress and heat shock, lead to the nuclear accumulation of KPNA2 in tumor cells [34,35,36]. Therefore, it is possible that high KPNA2 expression, observed during RT, causes poor responsiveness to cancer treatment; in these instances, alternative therapeutic options might be more advantageous than RT.

Here, we showed that KPNA2 was significantly increased by radiation in the human colorectal cancer cell lines HT29 and HCT116 (Figure 1). Cell proliferation and colony formation were impaired, and apoptosis was promoted in *KPNA2*-silenced cells exposed to IR, compared with irradiated cells (Figure 2). It has been reported that KPNA2 interacts with the NLS of many cargo proteins including Nijmegen breakage syndrome 1 (NBS1), octamer-binding transcription factor 4 (Oct4), nuclear factor kappa-light-chain-enhancer of activated B cells (NF-κB), c-Myc, p53, Chk2, BRCA1, Ras-related C3 botulinum toxin substrate 1 (RAC1), and p65 [12,16,20,37,38,39,40,41]. Tseng et al. demonstrated that the KPNA2-NBS1 interaction contributes to the nuclear translocation and nuclear focus formation of the NBS1 complex, enabling multiple tumor suppression functions upon exposure to radiation [40]. In addition, an earlier study has already demonstrated that two functional NLSs in BRCA1 interact with the importin-α subunit, and any defect in their interaction system could lead to a failure of BRCA1 translocation [42]. Numerous pieces of evidence suggest that the tumor suppressor BRCA1 plays a major role in cell cycle control and various DNA repair pathways, including homologous recombination, non-homologous end-joining, and base excision repair, and serves to maintain genomic fidelity [43]. Therefore, it can be hypothesized that BRCA1 may contribute to mitigate radiotoxicity and chromosomal instability through repair of clustered DNA lesions [26]. Since BRCA1 is a nuclear-cytoplasmic shuttling protein, targeting BRCA1 localization and shuttling is a novel strategy to enhance the cytotoxic response to DNA damage agents; BRCA1 contributes to DDR in the nucleus but activates cell death signals in the cytoplasm [44]. These data suggest that KPNA2 functions as a key transporter of DNA repair molecules required for the maintenance of genomic stability under DNA damaging conditions such as IR. This hypothesis was supported by the finding that KPNA2-BRCA1 interaction, as well as the expression of KPNA2 and BRCA1, was increased by IR (Figure 3). These findings support the fact that the nuclear localization of BRCA1 with high expression of KPNA2 can serve as a functional biomarker to predict proceeding DNA damage repair actively and the development of radioresistance in cancer cells. In contrast to the report that BRCA1 is exported from the nucleus as early as 4 h and persists 50 h after irradiation [45], we found nuclear accumulation of BRCA1 48 h after irradiation, along with residual levels of γH2AX. This discrepancy may be due to the differences in the extent of IR-induced DNA damage (dose and dose rate of radiation) in different cell types. BRCA1 is directly involved in the repair of DSBs, and three BRCA1 complexes are known to play important roles at different stages in DSB repair [46,47]. The partner proteins of these complexes are the phosphorylated abraxas, CtIP, and BACH1 [48,49]. In addition, BRCA1 associates and co-localizes with various DNA damage repair proteins such as MSH2, RAD51, ATM, BKM, and the MRE11–RAD50–NBS1 (BASC model) complex [50]. Therefore, the time-dependent function of BRCA1 and its interacting partner proteins involved in IR-induced DDR require further investigation.

As DNA damage can activate AMPK to promote metabolic homeostasis and survival, we examined the activation of AMPK, a cellular sensor of metabolic stress, in our system. As expected, both pAMPK and KPNA2 were increased by radiation, and an AMPK inhibitor attenuated KPNA2 and BRCA1 accumulation (Figure 4), indicating that AMPK is an upstream molecule that regulates KPNA2. In agreement with our findings, the activation of AMPK by polyamines promotes importin-α1-mediated nuclear import of human antigen R (HuR) [51]. AMPK is a target of drugs for metabolic syndrome and type-2 diabetes. Targeting AMPK might also be beneficial for cancer treatment. However, KPNA2 could be a promising alternative target for anticancer drugs. To confirm whether our findings were cell-type dependent, we tested human breast cancer cells under the same experimental conditions. The results of *KPNA2* silencing in MDA-MB231 cells were consistent with those in colorectal cancer cells, indicating that KPNA2 is a target to circumvent radioresistance (Figure 5).

In conclusion, we demonstrated that IR increases KPNA2 via AMPK activation. KPNA2 can bind to BRCA1 and transport it to the nucleus in response to IR, possibly contributing to DNA damage repair. Knockdown of *KPNA2* resulted in accumulation of BRCA1 in the cytoplasm, increased apoptosis, and sensitized the cells to radiation (Figure 5E). However, important questions remain to be addressed regarding the upstream signals of KPNA2 induction and KPNA2 function itself. Further investigations are required to determine whether KPNA2 is responsible for other genotoxic stress conditions, such as UV light exposure, oxidative stress, and treatment with chemotherapeutic agents, and which characteristic proteins are involved in DDR. In addition, it remains to be determined whether these results may be extrapolated to in vivo animal or clinical studies. Our findings provide new insights and suggest that knockdown of *KPNA2* effectively improves the therapeutic effects of RT in patients with radioresistant cancer cells. Therefore, KPNA2 may serve as a prognostic biomarker and therapeutic target for radiation therapy.

## 4. Materials and Methods

### 4.1. Cell Culture

The human colorectal cancer cell lines HT29 and HCT116 and human breast cancer cell line MDA-MB-231 were obtained from the American Type Culture Collection (ATCC, Manassas, VA, USA). The cells were grown in RPMI-1640 medium (HyClone, Logan, UT, USA) supplemented with 10% fetal bovine serum, 100 IU/mL penicillin, and 100 μg/mL streptomycin (Invitrogen, Carlsbad, CA, USA). Cells were maintained at 37 °C in a humidified atmosphere of 5% CO_2_.

### 4.2. siRNA Transfection

*KPNA2* RNA interference was performed using 19-base siRNA duplexes purchased from Genolution Pharmaceuticals Inc. (Seoul, Korea). The sequences of siRNA were as follows: 5′-GCCGUGACCAACUAUACCA-3′ and 5′-UGGUAUAGUUGGUCACGGC-3′. Non-targeting siRNA (Genolution Pharmaceuticals Inc.) was used as a negative control. Sub-confluent tumor cell lines were transfected with siRNA duplexes (5 nM) using Lipofectamine RNAiMAX (Invitrogen) according to the manufacturer’s recommendations.

### 4.3. Western Blot Analysis

Total proteins from cell lines were extracted in TNN buffer (50 mM Tris-Cl, pH 7.4; 1% NP-40; 150 mM NaCl; and 1 mM ethylenediaminetetraacetic acid [EDTA]) supplemented with protease inhibitors (1 mM phenylmethylsulfonyl fluoride (PMSF), 1 μg/mL aprotinin, 1 μg/mL leupeptin, and 1 mM Na_3_VO_4_) and quantified using the Bradford method. Protein samples (15 μg) were separated by sodium dodecyl sulfate/polyacrylamide gel electrophoresis and transferred to a nitrocellulose membrane. After blocking non-specific antibody binding sites, the membrane was incubated overnight at 4 °C with monoclonal antibody against pBRCA1 (9009S, Cell Signaling, Danvers, MA, USA), BRCA1 (sc-6954, Santa Cruz Biotechnology, Paso Robles, CA, USA), cleaved caspase-3 (9664, Cell Signaling), KPNA2 (sc-55537, Santa Cruz Biotechnology), PARP (sc-7150, Santa Cruz Biotechnology), pAMPK (2535S, Cell Signaling), β-actin (A5316, Sigma-Aldrich, St. Louis, MO, USA), and lamin A/C (ab108922, Abcam, Cambridge, MA, USA). All the antibodies were used at a dilution of 1:1000. After incubation with peroxidase-conjugated secondary antibodies at 37 °C for 1 h, the protein bands were visualized using enhanced chemiluminescence reagent (GE Healthcare Biosciences, Piscataway, NJ, USA) and detected using the Amersham Imager 680 (GE Healthcare Biosciences). β-actin and lamin A/C were used for normalization.

### 4.4. Cell Viability Assay

Cell viability was evaluated with the 3-(4,5-dimethylthiazol-2-yl)-2,5-diphenyltetrazolium bromide (MTT) colorimetric growth assay. Briefly, cells (1000 cells/well) were seeded in 96-well plates, treated with *KPNA2* siRNA (5 nM) for 24 h, and then exposed to 8 or 10 Gy of IR with a ^137^Cs gamma irradiator (LDI-KCCH, Seoul, Korea; dose rate 0.1 cGy/min). The cells were stained with MTT solution (5 mg/mL, Sigma-Aldrich) for 4 h at 37 °C at the indicated times. The culture medium was then removed, and 100 μL of dimethyl sulfoxide (Sigma-Aldrich) was added. The absorbance was measured at 540 nm using a microplate reader (Multiskan EX, Thermo Fisher Scientific, Waltham, MA, USA). All experiments were performed in triplicate.

### 4.5. Clonogenic Assay

Cell survival after irradiation was determined by a clonogenic assay. Briefly, cells were seeded on 60-mm tissue culture dishes at various densities and then treated with several doses of radiation. After 12–14 days, colonies were fixed and stained with 1% methylene blue (Sigma-Aldrich) in absolute methanol solution for 10 min. Colonies larger than 0.1 mm diameter were scored as surviving colonies. The experiment was performed in triplicate for each cell line.

### 4.6. Annexin V/Propidium Iodide Staining

Apoptosis was analyzed by flow cytometry using the annexin V and PI staining method. Following transfection and radiation, tumor cells were harvested washed with ice-cold phosphate-buffered saline (PBS) without Ca^2+^ or Mg^2+^ and resuspended in the binding buffer; 5 μL annexin V-allophycocyanin (APC; 20 μg/mL) and 5 μL PI (50 μg/mL) were then added. After incubation in the dark for 15 min, the samples were analyzed using a FACScaliber flow cytometer (BD Biosciences, San Diego, CA, USA) and the FlowJo software (v.10, Tree Star, Ashland, OR, USA).

### 4.7. Preparation of Cytoplasmic and Nuclear Fractions

After transfection, irradiated cells were briefly spun down in microtubes for 5 min at 1500 rpm in a centrifuge, gently resuspended in 200 μL hypotonic buffer (10 mM 4-(2-hydroxyethyl)-1-piperazineethanesulfonic acid [HEPES], pH 7.9; 1.5 mM MgCl_2_; and 10 mM KCl) and placed on ice for 15 min. Next, 10% NP-40 was added to a final concentration of 0.5% (10 μL). Samples were mixed by inversion and spun at 6500 rpm for 30 s. Supernatants (cytoplasmic fractions) were collected for processing. The pellet was gently resuspended in 40 μL hypotonic buffers and rotated for 20 min at 4 °C. These samples were spun at 13000 rpm for 20 min, and the supernatants (nuclear fraction) were collected. Both the cytoplasmic and nuclear fractions were quantified using the Bradford method, as recommended by the manufacturer’s instructions.

### 4.8. Comet Assay

The single-cell gel electrophoresis assay (Comet assay) was utilized to determine DNA damage [52,53]. Briefly, cells were suspended in cold PBS, and an aliquot of cells was added to 100 μL molten low-melting-point agarose maintained at 39 °C. An aliquot of 10 μL was immediately spread onto each comet slide at 37 °C. The slide was incubated at 4 °C for 10 min to accelerate gelling of the agarose and then transferred to cold lysis solution (100 mM EDTA, 2.5 M sodium chloride, 10 mM Tris-Cl (pH 8.4), and 10% DMSO with 1% Triton X-100) for 60 min at 4 °C. The slides were then subjected to electrophoresis with cold Tris/Borate/EDTA (TBE, pH 8.0) at 50 volts for 90 min. After electrophoresis, the slides were washed with deionized H_2_O and then stained with fluorescent RedSafe™ nucleic acid staining solution (iNtRON biotechnology, Sungnam, Korea) for 10 min. The slides were analyzed using an LSM710 confocal microscope (Zeiss, Jena, Germany) and the Axiovision 4.2 software (Zeiss). Quantitation of 100 nuclei from each gel was performed using the open-source software OpenComet (v1.3.1, www.cometbio.org). Upon electrophoresis, in contrast to undamaged DNA, the damaged DNA showed the characteristic long ‘comet tail’ structures.

### 4.9. Immunoprecipitation Assay

HCT116 and HT29 cells were transfected with *KPNA2* siRNA (5 nM) using Lipofectamine RNAiMAX for 24 h and then irradiated with 8 or 10 Gy. Forty-eight hours later, cell lysates were prepared with RIPA lysis buffer supplemented with protease inhibitors as described in Section 4.3, but without sodium dodecyl sulfate. Protein concentrations were determined by the Bradford method. The cell lysates (2 mg) were used for IP and incubated with anti-KPNA2 or anti-BRCA1 primary antibodies or IgG at 4 °C overnight. Next, cell lysates were mixed with Protein A/G PLUS-Agarose beads (Santa Cruz Biotechnology) for 1 h at 4 °C, followed by washing with lysis buffer three times. The eluate was subjected to immunoblotting with the indicated antibodies.

### 4.10. In Situ Proximity Ligation Assay

Direct protein–protein interaction was visualized by in situ PLA [54] using Duolink In Situ Red Starter Kit Mouse/Rabbit (Sigma-Aldrich). Non-transfected or transfected cells were irradiated, further incubated for 48 h, rapidly washed with ice-cold PBS, and then fixed at 20 °C for 10 min in 4% paraformaldehyde. Cells were rinsed three times for 3 min with PBS containing 0.1% Triton X-100 (Sigma-Aldrich) and then washed three times for 2 min with 0.05% Tween 20 in Tris-buffered saline (TBS) to allow permeabilization. Next, one droplet (40 μL) of Duolink II blocking solution was added to each coverslip, followed by incubation in a preheated humidity chamber for 30 min at 37 °C. The blocking solution was then tapped off, and cells were incubated overnight with primary antibodies (dilutions as indicated in the datasheets). The next day, Duolink anti-rabbit PLUS and anti-mouse MINUS secondary antibodies and the red detection reagent were used and the antibody incubation, ligation, amplification, and washing steps were performed according to the manufacturer’s instructions. Coverslips were then mounted using Duolink Mounting Media with 4′,6-diamidino-2-phenylindole (DAPI) and the images were acquired under an LSM710 confocal microscope (Zeiss, Jena, Germany) using the Axiovision 4.2 software (Zeiss) and ImageJ software (National Institutes of Health, Bethesda, MD, USA) for image processing and analysis.

### 4.11. Immunofluorescence Staining

Cells were fixed for 15 min with 2% paraformaldehyde and permeabilized with 0.5% Triton X-100 (Sigma-Aldrich). Incubation with a mouse monoclonal primary antibody specific for phosphorylation of H2AX (sc-517348, Santa Cruz Biotechnology) for DNA damage detection followed by incubation with an Alexa Fluor 488-labeled secondary antibody (Molecular Probes, Leiden, The Netherlands, 1:1000). For evaluation of cellular co-localization, a mouse monoclonal KPNA2 primary antibody and a rabbit monoclonal pBRCA1 primary antibody were used with an Alexa Fluor 488- and 594-labeled secondary antibody. Coverslips were then mounted using Duolink Mounting Media with 4′,6-diamidino-2-phenylindole (DAPI), and the images were acquired under an LSM880 confocal microscope (Zeiss) using the Zen 2.3 software (Zeiss) for image processing and analysis.

### 4.12. Statistical Analysis

Statistical analyses were performed using GraphPad software version 5 (GraphPad, La Jolla, CA, USA). All data are expressed as the mean ± the standard deviation (SD). Significant differences between the groups were determined by analysis of variance (ANOVA) and Tukey’s post hoc test. *P* values lower than 0.05 were considered statistically significant.
